# Structural and Functional Characterization of the Human Protein Kinase ASK1

**DOI:** 10.1016/j.str.2007.08.011

**Published:** 2007-10-16

**Authors:** Gabor Bunkoczi, Eidarus Salah, Panagis Filippakopoulos, Oleg Fedorov, Susanne Müller, Frank Sobott, Sirlester A. Parker, Haifeng Zhang, Wang Min, Benjamin E. Turk, Stefan Knapp

**Affiliations:** 1 University of Oxford, Structural Genomics Consortium, Botnar Research Centre, Oxford OX3 7LD, United Kingdom; 2 Department of Pharmacology, Yale University School of Medicine, New Haven, CT 06520, USA; 3 Department of Pathology, Yale University School of Medicine, New Haven, CT 06520, USA

**Keywords:** PROTEINS, SIGNALING

## Abstract

Apoptosis signal-regulating kinase 1 (ASK1) plays an essential role in stress and immune response and has been linked to the development of several diseases. Here, we present the structure of the human ASK1 catalytic domain in complex with staurosporine. Analytical ultracentrifugation (AUC) and crystallographic analysis showed that ASK1 forms a tight dimer (K_d_ ∼ 0.2 μM) interacting in a head-to-tail fashion. We found that the ASK1 phosphorylation motifs differ from known ASK1 phosphorylation sites but correspond well to autophosphorylation sites identified by mass spectrometry. Reporter gene assays showed that all three identified in vitro autophosphorylation sites (Thr813, Thr838, Thr842) regulate ASK1 signaling, but site-directed mutants showed catalytic activities similar to wild-type ASK1, suggesting a regulatory mechanism independent of ASK1 kinase activity. The determined high-resolution structure of ASK1 and identified ATP mimetic inhibitors will provide a first starting point for the further development of selective inhibitors.

## Introduction

Apoptosis signal-regulating kinase 1 (ASK1, also called MAP3K5) is a mitogen-activated protein kinase kinase kinase (MAP3K) that plays an essential role in cellular stress and the immune response (Matsukawa et al., 2004). ASK1 activates both the JNK and p38 pathways by direct phosphorylation of MAP kinase kinases (MKKs). ASK1 is activated by various stimuli, including lipopolysaccharide (LPS), oxidative stress (ROS), endoplasmatic reticulum (ER) stress, influx of calcium ions, cytokines (such as tumor necrosis factor α), Fas ligand, and GPCR agonists (Ichijo et al., 1997; Chen et al., 1999; McDonald et al., 2000). ASK1 knockout mice (ASK1^−/−^) show no overt macroscopic or microscopic phenotype but are resistant to LPS-induced septic shock, demonstrating the essential role of ASK1 in inflammatory signaling (Matsuzawa et al., 2005). Overexpression of ASK1 can induce apoptosis but depending on the cellular context may also promote differentiation and survival (Takizawa et al., 2002; Sayama et al., 2001).

ASK1 has been linked to several diseases and has been discussed as a target for pharmaceutical intervention (Hashimoto et al., 2003; Harada et al., 2006). For example, ASK1 has been implicated in polyglutamine (PolyQ) diseases, which include at least nine inherited neurodegenerative disorders: Huntington's disease (HD), spinobulbar muscular atrophy (SBMA), dentatorubral-pallidoluysian atrophy (DRPLA), six spinocerebellar ataxias, and SCA3/Machado-Joseph disease (MJD). During disease progression, polypeptides called PolyQ fragments accumulate as aggregates in the cytoplasm and/or nucleus and induce cellular stress that leads to neuronal cell death in an ASK1-dependent manner (Kariya et al., 2005; Nishitoh et al., 2002; Sekine et al., 2006). ASK1 is also a potential therapeutic target for treating malignant fibrous histiocytomas, a particularly aggressive form of undifferentiated liposarcoma (Chibon et al., 2004). Activation of ASK1 by reactive oxygen species is also a key mechanism for β-amyloid induced neurotoxicity in Alzheimer's disease (Hashimoto et al., 2003; Kadowaki et al., 2005; Song et al., 2003).

**Figure 1:**
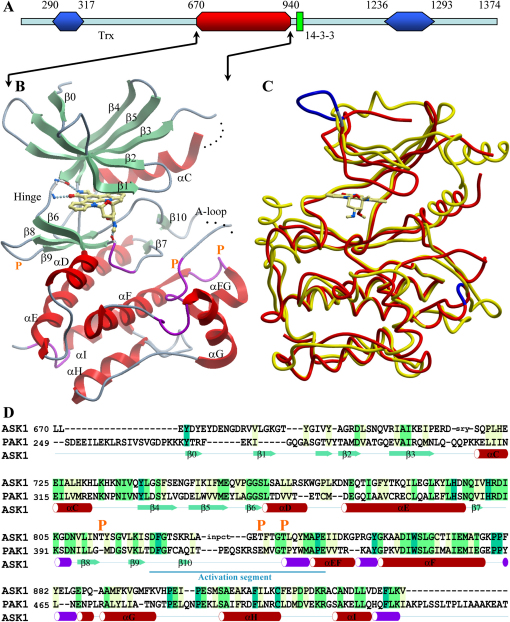
Overall Structures of ASK1 and Sequence Comparison to PAK1 (A) Domain architecture of ASK1. Coiled-coil domains are shown in blue, the kinase domain in red, and the 14-3-3 binding site in green. Predicted domain boundaries (residues) are given in the numbers above the sketch. (B) Ribbon diagram showing a structural overview. Secondary structure elements were determined with the program ICM Pro 3.4-8 (Molsoft, LLC) and have been labeled according to the nomenclature established for PKA (Knighton et al., 1991a). The helices are shown in red, β strands in green, and the 3_10_-helices in magenta. Disordered regions are indicated by dotted lines, and location of autophosphorylation sites are indicated by a yellow “P.” (C) Superimposition of ASK1 (red) with its closes structural neighbor PAK1 (yellow). (D) Structure-based sequence alignment of ASK1 with PAK1. Secondary structure elements of ASK1 are also shown and labeled. Autophosphorylation sites identified by mass spectrometry are indicated by “P.” Residues not included in the model due to disorder are indicated by lower case letters.

Human ASK1 is a polypeptide of 1,374 residues consisting of a central serine/threonine kinase domain and coiled-coil domains in both the N and C termini (Figure 1). ASK1 interacts with a large number of partners, forming a high molecular weight complex of approximately 1,500–2,000 kDa designated the ASK1 signalosome (Tobiume et al., 2002; Noguchi et al., 2005). In its resting state, ASK1 forms a homodimer stabilized by the C-terminal coiled-coil domain. The N-terminal domain is bound to the redox-regulatory protein thioredoxin (TRX), preventing its activation. Oxidative stress leads to disulfide bridge formation in TRX and dissociation from ASK1 (Saitoh et al., 1998; Liu and Min, 2002; Zhang et al., 2004). This triggers a conformational change in the ASK1 dimer leading to phosphorylation at the activation segment residue Thr838 by either autophosphorylation or transphosphorylation by an unidentified kinase (Tobiume et al., 2002).

Activated ASK1 directly phosphorylates MKK3/MKK6 and MKK4/MKK7, resulting in activation of the p38 and JNK MAPKs, respectively. A consensus docking site of 24 amino acids, called the DVD domain, is located in the C terminus of MKKs and has been proposed to interact with the kinase domain of ASK1, thus promoting the specific targeting of these downstream kinases (Takekawa et al., 2005).

Here, we present the high-resolution structure of the kinase domain of ASK1 in complex with the generic protein kinase inhibitor staurosporine. We show that the ASK1 catalytic domain is dimeric in solution and that it is an active kinase in its unphosphorylated state. We identified a consensus recognition sequence for ASK1 by peptide library screening that is in agreement with in vitro autophosphorylation sites identified by mass spectrometry. The high-resolution crystal structure together with the in vitro characterization of this enzyme provide a basis for the further understanding of ASK1 regulation and for the development of specific inhibitors for this interesting target.

## Results

### Overall Architecture

The ASK1 catalytic domain structure displays a typical protein kinase fold comprising the five β sheets and helix αC constituting the small lobe (residues 670–757) and a larger mainly alpha helical C-terminal lobe (residues 761–940) (Figure 1). The hinge region connecting the two domains lines the catalytic ATP binding site, which is occupied by the ATP competitive inhibitor staurosporine. The asymmetric unit harbors two ASK1 molecules, which can be superimposed with an rmsd of 0.6 Å and can therefore be considered structurally identical. Residues located in the loop regions at the beginning of αC (Asp715-Ser719) and the activation segment residues Gly831-Glu837 were not visible in the electron density and were assumed to be disordered. The N-terminus forms a short β sheet (β_0_), which is connected to the sheet β_1_ by an unusually large loop comprising residues 675–684. This large loop insertion may play a role in regulation of ATP binding since it determines the position of the phosphate binding loop, whereas the N-terminal sheet β_0_ extends the antiparallel sheet network in the smaller lobe, possibly adding stability to this domain.

ASK1 is only distantly related in sequence to kinases of known structure. Superimposition with the catalytic domain of its next structural neighbor PAK1 (PDB code: 1YHW; 29% sequence identity) revealed that the two kinases superimpose with an rmsd of about 2 Å (Figure 1C). However, structure determination by molecular replacement was challenging and required an improved search model built on the basis of SAD data collected from another crystal form (Table 1). The most striking difference between these two structures is that helix αC in ASK1 is moved further away from the active site. This has also been described for the MAP3K family member and the closest relative on the phylogenetic tree, TAK1 (PDB code: 2EVA). As a consequence of the outward shifted αC helix, the hydrogen bond between the conserved active site lysine (Lys709) and αC glutamate (Glu725) typically found in active kinases is not present in the ASK1 structure described here, and the domain therefore assumes a catalytically nonproductive conformation.

### Active Site and Binding of Staurosporine

**Figure 2:**
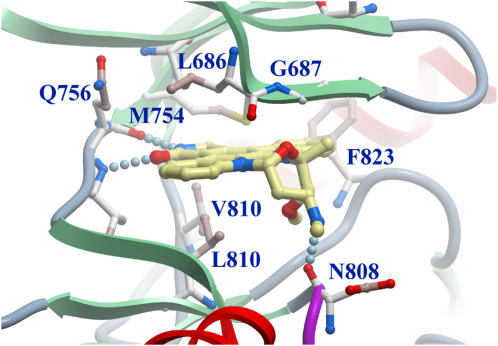
Binding of Stauroporine Main interacting active site residues with the ATP competitive inhibitor staurosporine are shown in ball-and-stick representation. Hydrogen bonds formed between the ligand and the protein are shown as dotted lines.

Staurosporine binds to the ATP binding site of ASK1 in a mode that has been described for a number of kinase-staurosporine complexes (Underwood et al., 2003; Bertrand et al., 2003) (Figure 2). Two hydrogen bonds anchor the lactam moiety to the hinge region and mimic the hydrogen-bonding pattern of the adenine base. The stauroporine ligand forms a third hydrogen bond with the backbone oxygen of the catalytic loop residue Asn808, an interaction that has been described previously (Lamers et al., 1999; Lawrie et al., 1997; Prade et al., 1997; Zhao et al., 2002; Zhu et al., 1999). The heterocyclic condensed five-ring system packs against the hydrophobic side chains of the ASK1 residues Leu686, Val694, and Ala707 on the N-terminal lobe side as well as Val738 and Leu810 on the C-terminal lobe side of the binding pocket. In addition to staurosporine, we identified a number of diverse inhibitors in the melting temperature screen performed on a library of 158 known kinase inhibitors (Table S1 and Figure S1 in the Supplemental Data available with this article online). The inhibitors have been used in cocrystallization experiments, but well-diffracting crystals were only obtained in the presence of staurosporine.

### Activation Segment

**Figure 3:**
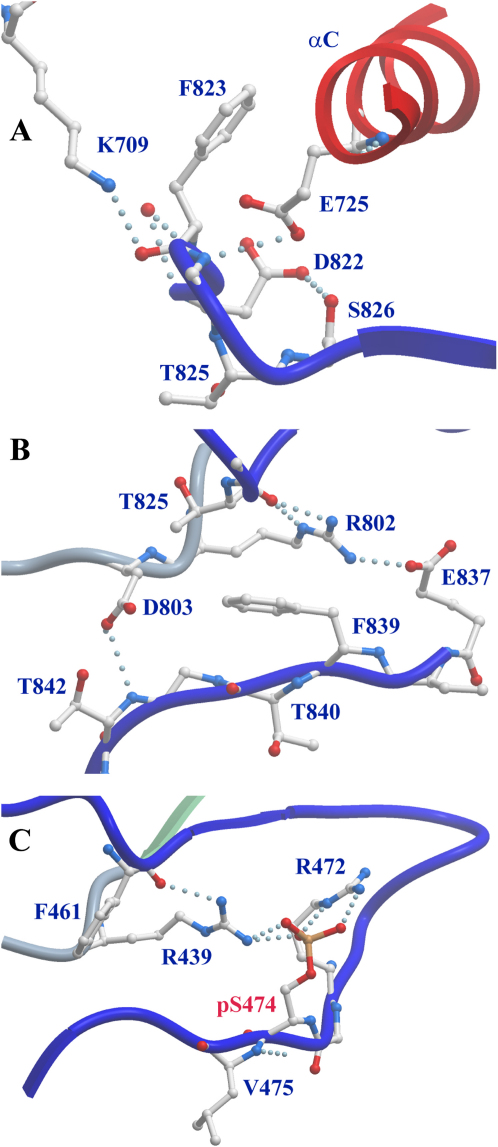
Activation Segment of ASK1 (A) Interaction between the helix αC and the DFG motif. Hydrogen bonds are shown as dotted lines. (B) Hydrogen bond network stabilizing N- and C-terminal portion of the activation segment and link the ASK1 activation segment to the catalytic loop residue Arg802. (C) Interaction stabilizing the activation segment in the active and phosphorylated kinase PAK4 (Eswaran et al., 2007).

Although not phosphorylated, the activation segment was well defined by electron density except for the unstructured tip of the activation loop (residues 832–836). Interestingly, the conserved αC glutamate (Glu725) forms a hydrogen bond with the main-chain nitrogen of the DFG motif residue Phe823, whereas the conserved active site lysine (Lys709), which usually forms a salt bridge with the αC glutamate in active kinases, also hydrogen bonds to the main chain of Phe823 (Figure 3A). The DFG aspartate (Asp822) forms a hydrogen bond with the activation segment residue Ser826. The observed noncatalytic conformation might be influenced by the presence of the bulky ATP competitive ligand staurosporine, which forms hydrophobic contacts with the DFG Phe823. In contrast, C-terminal to the DFG motif the activation segment is stabilized by interactions typically found in activated kinases. Comparison with the structure of phosphorylated, active PAK4 (Eswaran et al., 2007) revealed that the ASK1 activation segment residue Glu837 mimics the hydrogen-bond pattern formed by an activating phosphate moiety. In active kinases, phosphorylation at this position typically results in formation of a hydrogen bond network linking the catalytic loop arginine of the HRD motif (Arg802 in ASK1) with both termini of the activation segment (Lawrie et al., 1997). These interactions stabilize the activation segment in a conformation competent for substrate binding (Figures 3B and 3C). Activation segment mutants that lead to activation of kinases such as the S474E mutant in PAK1 (Lei et al., 2005), or activation segments of constitutively active wild-type kinases, mimic the hydrogen bond patterns formed by the activating phosphate moiety. Thus, the position and interactions formed by Glu837 in ASK1 suggest an activating and stabilizing function of this residue in unphosphorylated ASK1. Indeed, we observed that unphosphorylated ASK1 is active and autophosphorylates rapidly at three sites in vitro.

### ASK1 Catalytic Domain Dimerizes with a Head-to-Tail Orientation

**Figure 4:**
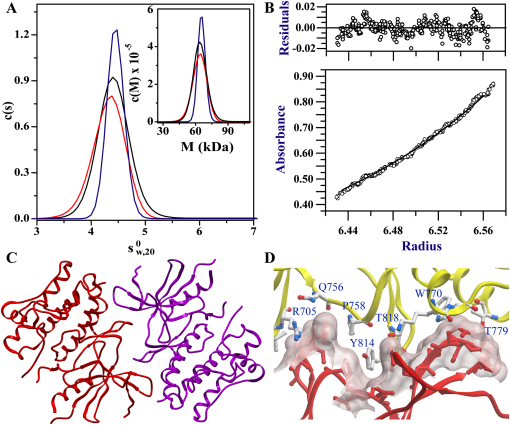
Dimerization of ASK1 Kinase Domain (A) Sedimentation velocity and equilibrium results of ASK1. Sedimentation velocity plot of the differential sedimentation coefficient distribution, c(s), versus the apparent sedimentation coefficient corrected to water at 20°C, *s_20,w_* of ASK1, together with the differential molecular weight distribution, c(M), versus molecular weight, M. Experiments were conducted with a protein concentration of 13 μM. Shown are Tri-phosphorylated ASK1 (red line), mixture of un- and monophosphorylated ASK1 (black line), and the mutant Thr813Ala (blue). (B) Sedimentation equilibrium experiment employing a rotor speed of 10,000 rpm. The solid line denotes a fitted curve resulting from global nonlinear regression analysis with a self-association model. The residuals for the fit are shown in the upper panel of the graph. The determined dissociation constant for the dimer was (K_D_) of 0.22 ± 0.2 μM. (C) Dimer interface spanning almost the entire side of ASK1 kinase domain. Both molecules interact in a head-to-tail orientation. (D) Details of interactions located in the dimer interface.

Full-length ASK1 has been described as a dimeric molecule stabilized in vivo by coiled-coil domain located C-terminal to the catalytic domain. Consequently, dimerization of the kinase domain was not an anticipated finding. Analysis of crystal packing revealed that the two ASK1 molecules in the asymmetric unit dimerize with their own symmetry equivalent. Both molecules bury a total surface area of about 1,997 Å^2^ consisting of 473 Å^2^ of hydrophobic and 1524 Å^2^ of hydrophilic surface. In order to verify the dimeric state of the ASK1 kinase domain in solution, we carried out analytical ultracentrifugation experiments (Figure 4). Sedimentation velocity measurements led to the determination of an apparent sedimentation coefficient corrected for water at 20°C, $s_{20,w}^{0}$, of 4.518 S, as well as the determination of a protein molecular weight of 66 kDa, which is in excellent agreement with the expected mass of an ASK1 catalytic domain dimer. Equilibrium experiments performed at three concentrations (c0, c0/2, and c0/4, where c0 is the concentration used in the velocity experiment) confirmed a stable dimeric state of the kinase domain in solution (Figure 4B). Nonlinear least square fit to a self association fitting model resulted in determination of a dissociation constant (K_D_) of 0.22 ± 0.2 μM.

The interaction between ASK1 monomers is based primarily on shape complementarity over a large surface area spanning almost the entire length of the protein. To achieve such a tight interaction, the molecules associate in a head-to-tail fashion with the N-terminal domain of one molecule interacting with the C-terminal domain of the other molecule and vice versa. The two catalytic sites are on the same side of the dimer near the equatorial plane and around 20 Å apart. Interaction between the molecules involves a number of direct hydrogen bonds (Leu700-Asn776', Asn702-Tyr783', Gln703-Thr779', and Arg705-Thr813' [and vice versa]), as well as water-mediated hydrogen bonds (Gln756-HOH-Tyr814') (the apostrophe denotes the symmetry equivalent molecule). In addition, multilayer π-stacking and hydrophobic interactions Arg705-Tyr814'-Pro758/Pro758'-Tyr814-Arg705' may also contribute to dimerization (Figures 4C and 4D and Table S3).

One of the identified phosphorylation sites (Thr813) is located in the dimer interface, and phosphorylation at this residue is likely to result in formation of hydrogen bonds to Arg705 located in the interacting protomer. We were therefore interested to explore if phosphorylation at Thr813 effects dimerization of ASK1. However, AUC data indicated that dephosphorylated ASK1 (mixture between de- and monophosphorylated ASK1) homogeneously triphosphorylated ASK1 as well as a T813A mutant formed all stable dimers in solution, and sedimentation equilibrium experiments revealed similar association behavior (Figure 4).

### Sequence Specificity of the ASK1 Substrate Binding Site

**Figure 5:**
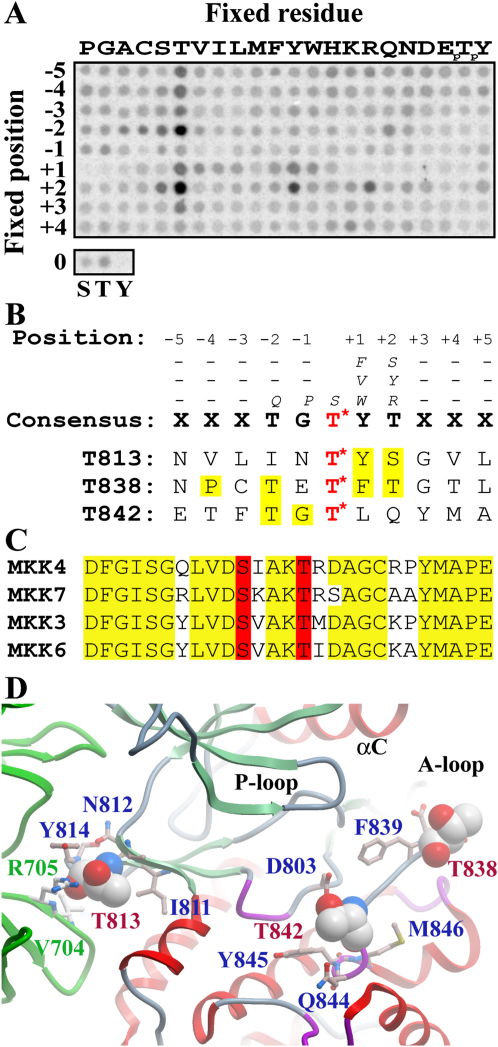
Substrate Specificity of ASK1 and Identification of Autophosphorylation Sites (A) Phosphorylation motifs for ASK1. Biotinylated peptides bearing the indicated residue at the indicated position relative to a central Ser/Thr phosphoacceptor site were subjected to phosphorylation by ASK1 with radiolabeled ATP. Aliquots of each reaction were subsequently spotted onto a streptavidin membrane, which was washed, dried, and exposed to a phosphor screen. Shown is a representative array from three separate experiments. Quantified spot intensities representing the average of the three runs are provided in Table S2. (B) Consensus sequence determined from the peptide array data. The consensus sequence is shown in bold and alternative residues are indicated at each position by smaller italic letters. The site of phosphorylation is indicated in red and by a star (^∗^). The three autophosphorylation sites are also shown, and residues matching the consensus sequence are highlighted with yellow boxes. (C) Activation segments of the known ASK1 substrates of the MKK family of kinases. Residues phosphorylated by activation are highlighted in red. (D) Location of the three autophosphorylation sites in ASK1. Phosphorylated residues are labeled in red and are shown as spheres, whereas neighboring residues are labeled in blue and are indicated in ball-and-stick representation. The interacting ASK1 dimer is shown in green.

In order to determine the sequence specificity of active site-mediated phosphorylation, we screened a peptide library with recombinant ASK1 (Figure 5A). The library was constructed to evaluate the contribution of all amino acid residues at each of nine positions (−5 to +4) surrounding a fixed central phosphoacceptor site (Hutti et al., 2004). The phosphorylation data revealed that ASK1 has a substantial preference for threonine over serine as a phospho-acceptor. ASK1 appears to be most selective at position +1 relative to the phosphorylation site, where it strongly prefers both aromatic and aliphatic hydrophobic residues in peptide substrates. We also observed strong phosphorylation of peptides bearing threonine residues in either the −2 or the +2 position. Because peptides in the library with fixed threonine residues can theoretically be phosphorylated at more than one site, these signals could reflect authentic selection for threonine at only one of the two positions. ASK1 also has secondary preferences for glutamine at the −2 position and for serine, arginine, and tyrosine at the +2 position. The kinase is not strongly selective at any of the other positions represented in the peptide library. The determined consensus motif is shown in Figure 5B.

### Autophosphorylation Sites of ASK1

The mass spectrum of the intact recombinant protein treated with ATP/Mg^2+^ clearly indicated the presence of at least three phosphorylation sites (Figure S2). The protein used for crystallization also contained small amounts of the monophosphorylated species arising during expression of the protein. The electron density map showed that Thr838 is partially phosphorylated, but due to the low occupancy of the phosphate moiety at this site, the phosphothreonine could not be convincingly modeled. An autophosphorylation site was identified on Thr813, which is located in the loop region connecting the sheets β8 and β9 pointing toward the C terminus of helix αD. The site is located at the protein surface very distant from the cofactor or substrate binding sites, but phosphorylation at Thr813 could impact lobe dynamics and thereby indirectly influence catalysis. Thr813 is also located in the dimer interface in close proximity to Arg705 of the interacting catalytic domain located in the loop between β_2_ and β_3_, but significant effects on dimerization were ruled out by AUC experiments carried out on an alanine mutant. In dimeric ASK1, Thr813 is not accessible, and the dissociation constant determined for the dimer association suggests that phosphorylation at this site is a slow process. However, structural changes in the ASK1 signalosome, which are likely to occur upon activation, might render this site more accessible.

A second site was identified at Thr838, which is located in the activation segment. In our structure, the side chain of Thr838 is oriented toward the solvent, and phosphorylation at this site is not expected to stabilize the activation segment in the absence of structural rearrangements. Furthermore, as described above, the neighboring residue Glu837 forms interactions typically formed by a phosphate moiety located at the corresponding position within the activation segment. However, Thr838 has been shown to play an essential role in activation of ASK1 by oxidative stress (Tobiume et al., 2002). A third phosphorylation site was identified at Thr842 located in close proximity (2.6 Å) to the catalytic aspertate (Asp803), and phosphorylation at Thr842 may lead to formation of hydrogen bonds with the catalytic loop lysine Lys805. All three sites, in particular Thr838, are in good accordance with our determined consensus peptide phosphorylation motif for ASK1 (Figure 5B) in contrast to known phosphorylation sites of downstream ASK1 substrates (Figure 5C). The location of the three autophosphorylation sites in our structural model is shown in Figure 5D.

### Role of ASK1 Catalytic Domain Phosphorylation Sites

**Figure 6:**
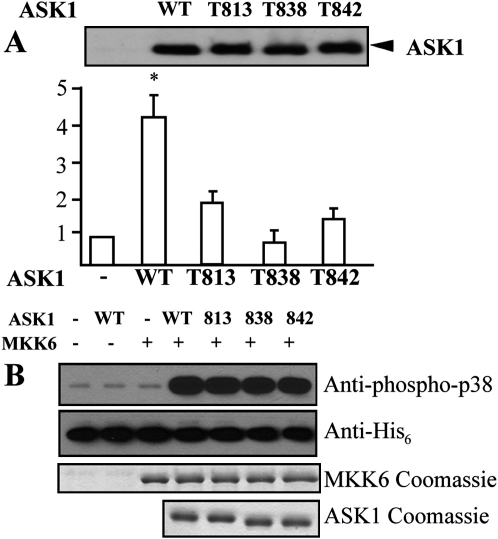
Effects of Mutation at T813A, T838A, and T842A on ASK1 Activity (A) 293T cells were transfected with various ASK1 mutants in the presence of an ASK1-JNK-dependent reporter gene. A renilla construct was cotransfected as an internal control. HIPK1-WT and T838A were used as controls. Both luciferase and renilla units were measured. Relative luciferase activities are presented from mean of duplicate samples by taking vector control as 1. Similar results were obtained from two additional experiments. Data are presented as mean of duplicates from two independent experiments. ASK1 protein expression was determined by Western blot (lower panel) with anti-HA-POD (anti-HA-conjugated peroxidase; Roche). (B) The ASK signaling pathway was reconstituted in vitro with recombinant ASK1 and its phosphorylation site mutants, MKK6 as well as p38. p38 phosphorylation was detected with an antibody specific phosphorylated p38. Corresponding Coomassie gels as well as a his-tag-specific antibody has been used to demonstrate identical loading concentrations of the samples.

To study the role of each of the identified autophosphorylation sites, we cloned site-directed alanine mutants into an expression vector and studied the effects of the mutations in transient transfection assays, monitoring JNK/p38 reporter gene activity. As expected from earlier studies, mutation of Thr838 drastically reduced reporter gene activity when compared to unstimulated control levels. Interestingly, mutation of the other two sites also provided a significant reduction in ASK1 function (Figure 6A), suggesting that autophosphorylation at the residues Thr842 and Thr813 regulates ASK1 signaling.

Furthermore, we studied the effect of the site-directed alanine mutants on kinase catalytic activity in vitro. Recombinant ASK1 mutants autophosphorylated rapidly and had specific activities that were comparable to wild-type ASK1 (data not shown). To test if the generated ASK1 mutants are also active on specific ASK1 substrates, we reconstituted a minimal ASK1 signaling cascade (ASK1-MKK6-p38) in vitro with recombinant MKK6 and His_6_-p38α by detecting p38 phosphorylation with an antibody specific for phosphorylated p38. Surprisingly, all three mutants showed catalytic activity in this in vitro phosphorylation experiment comparable to wild-type ASK1, suggesting that phosphorylation at the three catalytic domain autophosphorylation sites may be required for the recruitment of signaling partners and ASK1 substrates to this large signalosome rather than for regulation of catalytic activity.

## Discussion

ASK1 shares only moderate sequence homology with the catalytic domains of other human protein kinases. Apart from its closely related isoform ASK2 (MAP3K6) the next phylogenetic neighbors share sequence identities of only about 50% with the ASK1 catalytic domain. As a consequence, the structure of ASK1 determined in this study represents a chemically diverse kinase catalytic domain.

Recombinant ASK1 was found to be catalytically active and rapidly autophosphorylated at Thr813, Thr838, and Thr842 in vitro. Even though recombinant ASK1 is catalytically active, the structure of ASK1 was found to assume a catalytically not competent conformation as indicated by the distal position of αC from the active site, the conformation of the DFG motif that would not allow binding of a metal ion and by the partial disorder of the activation loop tip. Inactive conformations are quite frequently observed in active kinases and reflect the dynamic nature of the kinase catalytic domain (Eswaran et al., 2007). Reporter gene assays of site-directed mutants demonstrated that all three residues are important for ASK1 signaling in cells. For mouse ASK1, corresponding activation loop positions for Thr838 and Thr842 (Thr845 and Thr849) have been identified earlier as critical residues for ASK1 activity by alanine scanning mutagenesis of all possible activation loop phosphorylation sites (Tobiume et al., 2002). In addition to these autophosphorylation sites, phosphorylation of three Ser residues (Ser83, Ser967, and Ser1034) by other kinases also regulate ASK1 signaling activity (Zhang et al., 1999; Kim et al., 2001; Fujii et al., 2004; Zhang et al., 2005) and the recruitment of binding partners. The association of ASK1 with the two phosphatases PP5 and Cdc25A supports a pivotal role for phosphorylation in regulating ASK1 activity (Morita et al., 2001; Zou et al., 2001).

Thr842 forms a hydrogen bond with the catalytic aspartate residue, which is destroyed by mutating this residue to alanine. However, the recombinant protein is still active and phosphorylates specific substrates (e.g., MKK6) with similar activity than wild-type ASK1. Interestingly, the analogous position in the activation segment has also been described as a regulatory phosphorylation site for DAPK3 (Thr150) (Graves et al., 2005) and CHK2 (Thr387). Phosphorylation at this site has been shown to be critical for CHK2 activity, and the mutation Thr387Ala is unable to trigger the G1 checkpoint (Lee and Chung, 2001).

Surprisingly, the ASK1 activation loop conformation does not explain how phosphorylation on Thr838 influences ASK1 activity, and interestingly, the mutant Thr838Ala is catalytic active and recognizes both autophosphorylation sites as well as specific substrates with similar efficiency as wild-type ASK1 in vitro. Thus, it is likely that in the absence of Thr838 phosphorylation, the neighboring residue Glu837 mimics hydrogen bond patterns typically formed by an activating phosphate moiety (Nolen et al., 2004). In the structure determined here, the side chain of Thr838 is oriented toward the solvent suggesting that formation of new stabilizing interactions upon phosphorylation would require structural rearrangement of the activation segment.

Such a conformational change is likely to contribute to the peptide phosphorylation specificity that we observed for ASK1. The serine-threonine kinases PKA and PKB/Akt are phosphorylated at activation loop sites analogous to that of ASK1 Thr838 and like ASK1, have a preference for hydrophobic, particularly aromatic residues at the +1 position in their substrates. In these kinases, two residues form a hydrophobic pocket that accommodates residues found at the +1 position in substrates: the residue immediately upstream of the APE sequence that terminates the activation loop (analogous to Met846 of ASK1) and the residue immediately downstream of the phosphorylated Thr within the loop (analogous to Phe839 of ASK1) (Knighton et al., 1991b; Yang et al., 2002). In the present structure, Phe839 is pointed toward the interior of the protein and is not in close contact with Met846, and thus no +1 hydrophobic pocket is apparent. Formation of a hydrophobic site analogous to that found in PKA and PKB/Akt would require a portion of the activation loop, including Phe839, to rotate ∼120° relative to its position within the structure. This rearrangement could be triggered through phosphorylation of Thr383 and its consequent interaction with Arg802.

In contrast to the autophosphorylation sites, which closely match the determined consensus sequence, several of the ASK1 phosphorylation sites on downstream MKK substrates do not. Phosphorylation of these nonconsensus sites may be driven by complex formation between ASK1 and the MKKs, which could overcome a specific sequence requirement at the phosphorylation site. Alternatively, structural changes in the activation segment that may impact phosphorylation site selectivity could be induced by binding of an ASK1 substrate to the DVD docking site. Significant rearrangement of the activation segment has recently been reported for the MAPK ERK2 upon engagement of a docking site (Zhou et al., 2006). In MAPKs, D motif docking peptides bind at analogous sites to p38α and JNK1 and induce conformational changes unique to each kinase (Chang et al., 2002; Heo et al., 2004). In ERK2, binding of the docking peptide results in a new conformation of the activation loop, while in p38α and JNK1 docking interactions induce disorder in the activation segment. Such allosteric effects associated with binding of docking peptides may provide a general mechanism for increasing signal flux and specificity in phosphorylation cascades, by inducing a conformation that promotes phosphorylation by the upstream kinase.

This study revealed that the catalytic domain of ASK1 also dimerizes independently of C-terminal coiled-coil domains. ASK1 has been reported to hetero-oligomerize with the closely related protein ASK2 (MAP3K6) (Takeda et al., 2007). All autophosphorylation sites as well as residues located in the dimer interface are conserved between these two closely related kinases suggesting that heterodimers of ASK1 and ASK2 may also arrange in a similar fashion. Moreover, ASK2 has been reported to directly activate ASK1 by phosphorylation, suggesting a mechanism of trans-activation within the ASK signalosome (Takeda et al., 2007). The head-to-tail orientation of the ASK1 kinase domain shows homo or hetero ASK1/2 oligomers aligned in a similar orientation as the full-length proteins and imply that the kinase domain itself makes an important contribution to formation of the dimer in addition to the C-terminal coiled-coil domain. However, more structural studies on complexes of ASK1 with its signaling partners and ideally the structure of full-length ASK1 would be necessary to understand the molecular details of the complex regulation of this kinase.

ASK1 has been suggested to be a potential target for the treatment of polyglutamine (polyQ) diseases (Hashimoto et al., 2003; Harada et al., 2006) and is also a potential therapeutic target for malignant fibrous histiocytomas, (Chibon et al., 2004). In addition, it has also been shown that activation of ASK1 by ROS is a key mechanism for amyloid β-induced neurotoxicity in Alzheimer's disease (Hashimoto et al., 2003; Kadowaki et al., 2005; Song et al., 2003). Furthermore, ASK1 is an essential regulator of innate immunity, and selective inhibitors would be interesting reagents for the development of clinical anti-inflammatory agents to treat septic shock. The structures presented here as well as the inhibitors identified will enable future structure-based development of selective inhibitors for this kinase.

## Experimental Procedures

### Cloning, Expression, and Purification

Human ASK1 (residues 659–951; gi|5174547) was subcloned into the T7 expression vector SGC-pLIC. The protein was expressed as an N-terminally His6-tagged fusion protein with a tobacco etch virus (TEV) protease cleavage site by using *Escherichia coli* BL21(DE3).

A 10 ml overnight culture was used to inoculate 1 l of Terrific Broth media containing 100 μg/ml kanamycin. Cultures were grown at 37°C until the OD_600_ reached ∼2.0. After that, the temperature was adjusted to 25°C, and expression was induced for 18 hr with 1 mM IPTG. Cells were centrifuged and pellets were resuspended in binding buffer (50 mM HEPES [pH 7.5], 300 mM NaCl, 20 mM imidazole including a protease inhibitor cocktail [Complete, Roche]) and lysed with a high-pressure homogenizer. The lysate was cleared by centrifugation, and the protein was purified by Ni-affinity chromatography with a wash buffer (50 mM HEPES [pH 7.5], 1 M NaCl, 20 mM Imidazole) and an elution buffer of the same composition but containing 250 mM imidazole. The eluted protein was treated with lambda and alkaline phosphatase together with TEV protease for 12 hr at 4°C to remove phosphorylation and the His6-tag, respectively. The eluted fraction was further purified by gel filtration chromatography with Superdex S75 (60 × 1 cm) column equilibrated in 10 mM HEPES (pH 7.5), 500 mM NaCl, 5% (w/v) glycerol. DTT was added to the protein sample to a final concentration of 10 mM, and the protein was concentrated to 13 mg/ml. Purity and integrity of ASK1 were confirmed by SDS/PAGE and electrospray ionization time-of-flight mass spectrometry (Aglient LC-ESI TOF).

### Analytical Ultracentrifugation

Sedimentation velocity experiments were carried out on a Beckman XL-I Analytical Ultracentrifuge equipped with a Ti-50 rotor and cells with double-sector centerpieces. Protein samples were studied at a concentration of 13 μM in 10 mM HEPES (pH 7.4) (at 25°C), 120 mM NaCl, 0.25% glycerol at 4°C, employing a rotor speed of 40,000 rpm. Radial absorbance scans were collected at a wavelength of 280 nm in 1 min intervals. Data were analyzed with SEDFIT (Schuck, 2000) calculating c(s) distributions. The software package SEDNTERP was used in order to normalize the obtained sedimentation coefficient values to the corresponding values in water at 20°C, $s_{20,w}^{0}$, taking into account the solvent density (*ρ_o_* = 1.00503 g/ml), viscosity (*η_o_* = 1.5862 × 10^−2^), and the protein partial specific volume (0.7309 ml/g). Sedimentation equilibrium experiments were performed with a six-sector centerpiece and protein concentrations of 6.5, 11.9, and 25.2 μM. Equilibrium was obtained after 24 hr at each of the two centrifugation speeds (10,000 rpm and 15,000 rpm). Baseline offsets were determined by using a meniscus depletion run. Sedimentation equilibrium data were evaluated by using Ultraspin.

### Determination of Peptide Phosphorylation Specificity

Phosphorylation motifs for ASK1 kinases were determined by using a positional scanning peptide library approach essentially as described before (Hutti et al., 2004). Reactions were carried out in multiwell plates in 50 mM HEPES (pH 7.4), 10 mM MgCl_2_, 1 mM DTT, 0.1% Tween 20, 100 μM ATP (including 0.3 μCi/μl γ-[^33^P]-ATP), 50 μM peptide substrate, and 50 μg/ml unphosphorylated ASK1 for 2 hr at 30°C. Peptide substrates had the general sequence YAXXXXX-S/T-XXXXAGKK(biotin), where S/T represents an even mixture of serine and threonine, K(biotin) is ɛ-(biotinamidocaproyl)lysine, and X is a roughly equimolar mixture of the 17 amino acids excluding cysteine, serine, and threonine. Each well contained a distinct peptide in which one of the X positions was replaced with 1 of 22 residues (one of the unmodified proteogenic amino acids exlcuding pSer and pThr). In addition, three additional wells were included that contained either no peptide, the peptide YAXXXXX-S-XXXXAGKK(biotin), or the peptide YAXXXXX-T-XXXXAGKK(biotin), to test phosphoacceptor residue preference. At the end of the incubation time, aliquots of each reaction were spotted onto streptavidin membrane, which was processed as described by Hutti and coworkers (Hutti et al., 2004).

### Phosphorylation Site Mapping

The protein was denatured by boiling, cysteine residues were reductively alkylated, and then a tryptic digest was performed overnight (100:1 protein/enzyme ratio). The peptides were separated on a Dionex 3000 nano-LC system with a C18 Pepmap column by using a water/acetonitrile gradient with 0.1% formic acid and analyzed with a Bruker HCT Ultra ion trap in MS/MS mode. Alternating fragmentation cycles were performed in data-dependent MS/MS by using collision-induced dissociation (CID) and electron transfer dissociation (ETD), the data were submitted to Mascot searches (http://www.matrixscience.com/) separately, and the information from the two alternative approaches was combined.

### Plasmid Construction for Transfection Studies

ASK1-WT and ASK1-T838 (human; T845 for murine) were described previously (Zhang et al., 2005). ASK1 mutants (T813A and T842A) were constructed by site-directed mutagenesis with a Quickchange site-directed mutagenesis kit (Stratagene) according to the protocol of the manufacturer. Transfection of 293T cells were performed by Lipofectamine (Invitrogen). Luciferase activity followed by renilla activity was measured twice in duplicate with a Berthold luminometer. All data were normalized as relative luciferase light units/renilla unit.

### Immunoblotting

Cell lysates were subjected to SDS-PAGE followed by immunoblot (IB) with a specific antibody (e.g., anti-HA) followed by HRP-conjugated anti-mouse secondary antibody. Protein was detected by chemiluminescence with an ECL kit according to the instructions of the manufacturer (Amersham Life Science).

### Crystallization, Data Collection, and Processing

Initial screens were set up with ASK1 kinase domain and several high-affinity small-molecular inhibitors. Crystals appeared in several conditions when 1 mM staurosporine was added to the protein. Screen conditions that yielded crystals all contained 20%–25% PEG 4000 or PEG 6000, 0.2 M salt, and had a pH around 6.0 (buffered or unbuffered). After optimisation, diffraction quality needle-like crystals were obtained from 25% PEG3350, 0.17 M (NH_4_)_2_SO_4_, and 15% glycerol with average dimensions 0.2 × 0.05 × 0.05 mm^3^ and were mounted with a loop and flash frozen by plunging into liquid nitrogen. Datasets were collected at the PXII beamline at the Swiss Light Source with a MAR225 detector at 0.972 Å.

Selenomethionine-labeled protein did not yield crystals using the same crystallization condition, and initial screening was repeated. Eventually, needle-like crystals were obtained from 18% PEG6000 and 0.1 M H_3_Cit/Na_3_Cit (pH 4.9). A SAD dataset was collected at the PXII beamline at the SLS by using the selenium peak wavelength (0.9789 Å, determined from a fluoresence scan). Diffraction images were processed with XDS (Kabsch, 2001) different scans were scaled together with XSCALE (Kabsch, 2001). Data collection statistics are shown in Table 1.

### Structure Solution and Refinement

Although molecular replacement solutions could be obtained by employing either an ensemble of most similar structures (29%–31% sequence identity, including only the C-terminal domain) or a homology model as search model, refinement was unstable and diverged. Unfortunately, the anomalous signal in the selenomethione-labeled protein was too weak to result in structure solution on its own. Although selenium positions could be located from the anomalous data with SHELXD (Sheldrick et al., 2001), no interpretable map was obtained.

Initially, the homology model that was used in molecular replacement of the unlabeled protein was used in Phaser (McCoy et al., 2005), and a molecular replacement solution for the selenomethionine crystal was obtained. This model was refined in refmac5 (Murshudov et al., 1997), and phase restraints (phase probability distribution was obtained by SHARP [La Fortelle and Bricogne, 1997] from Se positions determined by SHELXD and expressed as Hendrickson-Lattman coefficients) were vital for keeping refinement stable. After several rounds of manual rebuilding, the model improved significantly and could be used for molecular replacement of the native dataset. Phaser located two molecules in the asymmetric unit, revealing that solutions obtained previously were incomplete and missing the second molecule. Multicrystal averaging, enabled by the fact that crystals from the unlabelled and selenomethionine-labeled protein were nonisomorphous and had a different cell and space group, was performed and resulted in a clear electron density map that was manually traced. Refinement was completed in refmac5 and converged to an R/R_free_ of 0.198/0.251. The resulting model was deposited with the Protein Data Bank under accession ID 2CLQ.

## Figures and Tables

**Table 1:** Data Collection and Refinement Statistics

	Native	Selenomethionine
Space group	P6_5_22	P4_1_2_1_2
Cell (Å)	a = b = 78.16; c = 423.28	a = b = 92.58; c = 85.19
Number of reflections	536667	116678
Number of unique data	35817	8659
Resolution (Å)^a^	47.0–2.30 (2.4–2.3)	46.3–2.90 (3.0–2.9)
Completeness^a^	99.3% (93.9%)	99.6% (96.7%)
I/σ(I)^a^	17.7 (1.9)	19.5 (2.8)
R_int_^a^	0.0991 (0.5924)	0.1047 (0.6648)
R_work_/R_free_	0.204/0.257	-
Rmsd bond length (Å)	0.014	-
Rmsd bond angle	1.52°	-
B-factors (overall) (mainchain, sidechain, solvent)	38.5 (38.4, 39.8, 33.6)	-
Ramachandran	Most favored: 91.6%; additionally allowed: 8.2%; generously allowed: 0.2%; disallowed: 0%	

a Values in brackets indicate values in the highest resolution shell.

## Accession Numbers

The crystal structure reported in this paper has been deposited in the Protein Data Bank, http://www.pdb.org/ (PDB ID code: 2CLQ).

## Supplemental Data

Document S1. Two Figures and Three Tables
