# Supplementary material for: Structural and Functional Characterization of the Human Protein Kinase ASK1
Source: Structure. 2007 Oct 16;15(10):1215–26. doi: 10.1016/j.str.2007.08.011 (PMC2100151; doi:10.1016/j.str.2007.08.011)
Supplement: Document S1. Two Figures and Three Tables [file mmc1.pdf]

Structure *15*

Supplemental Data

Structural and Functional Characterization

# of the Human Protein Kinase ASK1

Gabor Bunkoczi, Eidarus Salah, Panagis Filippakopoulos, Oleg Fedorov, Susanne Müller, Frank Sobott, Sirlester A. Parker, Haifeng Zhang, Wang Min, Benjamin E. Turk, and Stefan Knapp

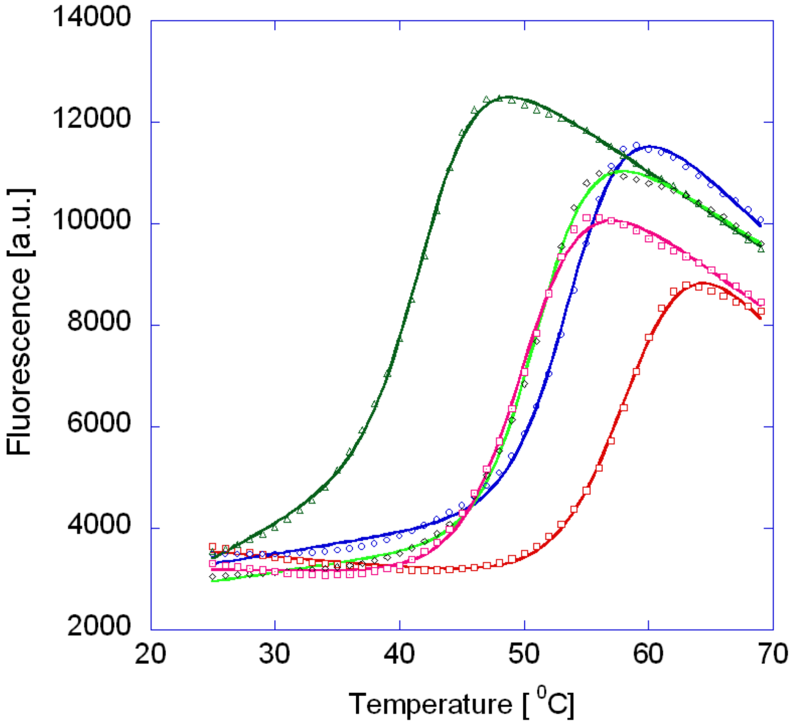

Figure S1. Stabilization of ASK1 by a Selection of ATP Mimetic Inhibitors Shown are ASK1 in the absence of inhibitors (olive line) as well as in the presence of 10 mM Staurosporine (red line), Hymenialdisine (blue line), JAK Inhibitor I (pink line), and SP600125 (light green line). The temperature stabilization in the presence of the identified ligands as well as the structure of the compound is summarized in Table S1.

Table S1. Inhibitors Identified Using Tm Shift Assays

Compound name	Calbiochem ID	Structure	T_m shift (°C)	Comment
**Staurosporine**	569397	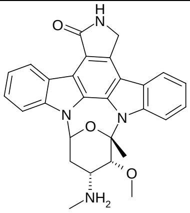	16.3	Non-selective kinase inhibitor
**CDK1/2 Inhibitor**	217714	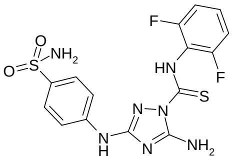	16.9	Cell-permeable anti-proliferative inhibitor developed for CDK1 and CDK2[1]
**Hymenialdisine**	400085	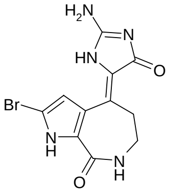	10.5	Cell-permeable, anti-inflammatory inhibitor of MEK1, CDK's and CK1[2]
**JAK Inhibitor I**	420099	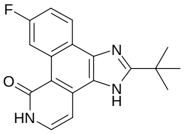	8.9	Cell-permeable potent inhibitor of JAK kinases and Tyk2[4].
**SP600125**	420119	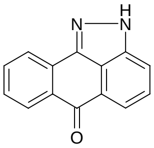	8.9	Cell-permeable potent inhibitor of JNK[5]

[1]Lin, R., et al. 2005.*J. Med. Chem.* **48,** 4208.
[2]Biernat, J., et al. 2002. *Mol. Cell Biol.* **13,** 4013. Tasdemir, D., et al. 2002. *J. Med. Chem.* **45,** 529. Curman, D., et al. 2001. *J. Biol. Chem.* **276,** 17914. Meijer, L., et al. 2000. *Chem. Biol.* **7,** 51. Breton, J.J., et al. 1997. *J. Pharmacol. Exp. Ther.* **282,** 459.
[3]Lai, J.Y.Q., et al. 2003. *Bioorg. Med. Chem. Lett.* **13,** 3111.
[4]Pedranzini, L., et al. 2006. *Cancer Res.* **66,** 9714. Lucet, I.S., et al. 2005. *Blood* **107,** 176. Thompson, J.E., et al. 2002. *Bioorg. Med. Chem. Lett.* **12,** 1219.
[5]Shin, M., and Boyd, Y.D. 2002. *Biochim. Biophys. Acta* **1589,** 311. Bennett, B.L., et al. 2001. *Proc. Natl. Acad. Sci. USA* **98,** 13681. Han, Z., et al. 2001. *J. Clin. Invest.* **108,** 73.

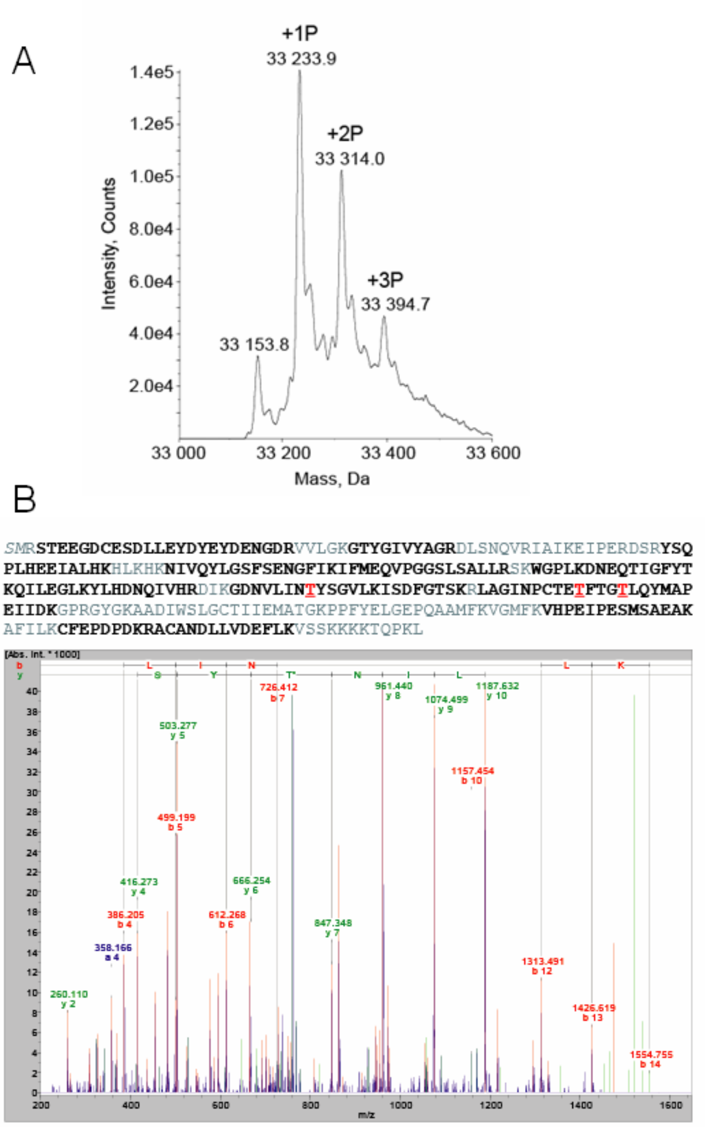

Figure S2. Auto-Phosphorylation of ASK1

A: ESI-MS spectrum of recombinant ASK1 after autophosphorylation. B: Sequence coverage and sequencing of peptides. Highlighted residues have been identified by nano-LC ion trap MS/MS in tryptic digests. Identified phosphorylated residues are highlighted in red.

Table S2. Quantified Peptide Array Data for ASK1 Phosphorylation Specificity

	-5	-4	-3	-2	-1	+1	+2	+3	+4
**P**	1.10	1.47	1.20	1.64	1.46	0.37	0.38	0.83	1.01
**G**	1.37	1.07	0.97	0.64	2.43	0.39	0.28	0.56	0.78
**A**	0.83	0.88	0.94	1.72	1.48	0.17	0.44	0.90	1.14
**C**	0.91	1.17	0.91	1.33	0.56	0.82	0.56	1.48	0.66
**S**	0.66	1.27	1.31	1.70	1.32	0.61	1.75	2.28	0.88
**T**	2.89	2.86	2.70	5.91	1.77	3.61	6.75	2.75	2.45
**V**	0.77	0.56	0.55	0.64	0.27	2.14	0.29	1.01	0.83
**I**	0.74	0.90	0.47	0.55	0.09	1.63	0.27	0.72	0.86
**L**	0.59	0.71	0.69	0.59	0.67	1.06	0.50	0.83	0.75
**M**	0.71	0.59	1.02	0.55	1.27	0.67	0.51	0.89	0.68
**F**	0.75	0.71	0.87	0.35	0.94	1.84	0.75	0.82	0.79
**Y**	0.72	0.89	0.98	0.61	1.33	2.74	2.45	0.89	0.94
**W**	0.76	0.94	0.96	0.30	0.51	1.47	0.65	0.64	0.83
**H**	1.38	0.81	1.05	0.64	0.87	0.94	0.55	0.82	1.17
**K**	0.89	0.76	0.83	0.33	0.93	0.23	0.85	1.10	1.75
**R**	1.23	0.75	0.78	0.40	1.05	0.23	1.39	1.11	1.17
**Q**	0.84	0.95	1.10	1.04	0.87	0.26	0.41	0.52	0.81
**N**	1.09	1.02	1.22	0.70	1.01	0.30	0.48	0.51	0.95
**D**	0.87	0.95	0.84	0.23	0.42	0.16	0.42	0.59	0.78
**E**	0.89	0.86	0.63	0.36	0.38	0.37	0.31	0.74	0.78
**pT**	1.03	0.66	0.53	0.22	0.79	0.14	0.22	0.36	0.53
**pY**	1.07	0.76	0.64	0.08	1.30	0.77	0.40	0.33	0.73

Data shown are the average of three separate determinations, each normalized so that the average value within a position is 1. Spots on the array were quantified using ImageQuant software.

Table S3. Details of ASK1 Dimer Interface

Property	Molecule A
**Number of Atoms**	
Interface (%)	111 (5.7%)
Surface (%)	1108 (56.6%)
Total (%)	1959 (100 %)
**Number of residues**	
Interface (%)	26 (10.0 %)
Surface (%)	237 (91.5%)
Total (%)	259 (100 %)
**Solvent-accessible Area ($Å^2$)**	
Interface/per monomer (%)	983.2 (7.7 %)
Total (%)	12838.9 (100%)

Hydrogen bonds	Molecule A	Distance [Å]	Molecule B
1	THR779 [OG1]	3.3	LEU700 [O]
2	ASN776 [ND2]	2.7	LEU700 [O]
3	THR779 [OG1]	2.9	SER701 [O]
4	TYR783 [OH2]	2.6	ASN702 [O]
5	ARG698 [NH2]	3.8	LYS769 [O]
6	GLN703 [NE2]	3.1	THR779 [OG1]
7	ARG705 [N]	2.8	THR813 [O]
8	ARG705 [NE]	3.1	THR813 [OG1]
9	LEU700 [O]	3.3	THR779 [OG1]
10	LEU700 [O]	2.7	ASN776 [ND2]
11	SER701 [O]	2.9	THR779 [OG1]
12	ASN702 [O]	2.6	TYR783 [OH2]
13	LYS769 [O]	3.8	ARG698 [NH2]
14	THR779 [OG1]	3.1	GLN703 [NE2]
15	THR813 [O]	2.8	ARG705 [N]
16	THR813 [OG1]	3.1	ARG705 [NE]
